# Growth Suppression in Lung Cancer Cells Harboring EGFR-C797S Mutation by Quercetin

**DOI:** 10.3390/biom11091271

**Published:** 2021-08-25

**Authors:** Kuo-Yen Huang, Tong-Hong Wang, Chin-Chuan Chen, Yann-Lii Leu, Hsin-Jung Li, Cai-Ling Jhong, Chi-Yuan Chen

**Affiliations:** 1 Department and Graduate Institute of Microbiology and Immunology, National Defense Medical Center, Taipei 11490, Taiwan; kyhuang0222@gmail.com; 2 Graduate Institute of Health Industry Technology and Research Center for Chinese Herbal Medicine, College of Human Ecology, Chang Gung University of Science and Technology, Taoyuan 33303, Taiwan; cellww@cgmh.org.tw (T.-H.W.); zpu8250upz@gmail.com (C.-L.J.); 3 Tissue Bank, Chang Gung Memorial Hospital at Linkou, Taoyuan 333, Taiwan; chinchuan@mail.cgu.edu.tw (C.-C.C.); ylleu@mail.cgu.edu.tw (Y.-L.L.); 4 Graduate Institute of Natural Products, Chang Gung University, Taoyuan 33303, Taiwan; 5 Institute of Cellular and Organismic Biology, Academia Sinica, Taipei 115, Taiwan; hsinjung0403@gmail.com

**Keywords:** AXL, EGFR C797S, lung cancer, quercetin, TKI resistance

## Abstract

Epidermal growth factor receptor (EGFR) tyrosine kinase inhibitors (TKIs) are approved treatments for non-small-cell lung cancer (NSCLC) patients harboring activating EGFR mutations. The EGFR C797S mutation is one of the known acquired-resistance mutations to the latest third-generation TKIs. At present, there are no clear options for treating patients who acquire resistance to third-generation TKIs. The acquisition of the EGFR C797S mutation was shown to upregulate the expression of AXL, a receptor tyrosine kinase of the TAM (TYRO3-AXL-MER) family, and the suppression of AXL is effective in reducing the growth of NSCLC cells harboring EGFR C797S. As quercetin was recently shown to inhibit AXL, quercetin may be effective in treating NSCLC cells harboring the EGFR C797S mutation. In this work, the cytotoxic effects of quercetin and its ability to inhibit tumor growth were examined in TKI-resistant NSCLC cells harboring the EGFR C797S mutation. We demonstrated that quercetin exhibited potent cytotoxic effects on NSCLC cells harboring the EGFR C797S mutation by inhibiting AXL and inducing apoptosis. Quercetin inhibited the tumor growth of xenografted NSCLC cells harboring the EGFR C797S mutation and appeared to act synergistically with brigatinib to inhibit of tumor growth in vivo. In summary, herein, we revealed that quercetin is an effective inhibitor for the treatment of non-small-cell lung cancer harboring the EGFR C797S mutation.

## 1. Introduction

Lung cancer remains the leading cause of cancer-related death worldwide, and non-small-cell lung cancer (NSCLC) is the most common type [1]. Targeted therapy designed to circumvent the epidermal growth factor receptor (EGFR) using tyrosine kinase inhibitors (TKIs) is a standard treatment for NSCLC patients harboring activating EGFR mutations [2]. Activating mutations in EGFR occur in 10–20% of Caucasian patients and in 30–40% of East Asian patients with NSCLC [3]. First- and second-generation TKIs are reversible or irreversible inhibitors that interact with the ATP-binding sites in the EGFR kinase domain, thus blocking the downstream signaling of EGFR. However, most patients develop TKI resistance within one year, most commonly due to the EGFR T790M mutation [4,5]. The third-generation TKI AZD9291 is highly active against the T790M mutation in NSCLC. However, its efficacy can be compromised by the EGFR C797S mutation, in which the key drug-interacting cysteine residue is replaced by serine [6]. Approximately 20–40% of TKI-resistant patients acquire the most common EGFR mutations, L858R/T790M/C797S and Del19/T790M/C797S. In an attempt to develop a treatment strategy to overcome the acquired EGFR C797S mutation, we recently showed that the EGFR C797S mutation is associated with the elevated expression of AXL, a receptor tyrosine kinase of the TAM (TYRO3-AXL-MER) family, and that the inhibition of AXL is effective in slowing the growth of NSCLC cells harboring EGFR C797S [7].

Quercetin, a bioflavonoid broadly distributed in plants, was shown to exhibit significant antitumor activity by modulating various cellular targets, leading to apoptosis induction, the suppression of proliferation, and the inhibition of metastasis [8]. Numerous studies have reported that quercetin induces apoptosis and inhibits the proliferation, migration, and invasion of NSCLC cells harboring wild-type EGFR [9,10,11,12,13,14,15], although it is not known whether TKI-resistant NSCLC cells are sensitive to quercetin. Recently, quercetin was reported to induce apoptosis by inhibiting AXL in glioblastoma cells [16]. The finding that quercetin inhibits AXL expression suggested that quercetin may be effective in treating NSCLC cells harboring the EGFR C797S mutation. In this work, we examined the antitumor activity of quercetin against TKI-resistant NSCLC cells cultured in vitro and in xenograft models in nude mice.

## 2. Materials and Methods

### 2.1. Culture Media, Reagents, and Antibodies

Fetal bovine serum and culture media were purchased from Life Technologies (Grand Island, NY, USA). All antibodies were purchased from commercial sources, as indicated below. Cell Signaling Technology (Danvers, MA, USA): anti-phospho-EGFR (Y1068), anti-phospho-AXL (Y702), anti-AXL, anti-phospho-AKT (S473), anti-phospho-ERK (T202/Y204), and anti-PARP (# 9541). Santa Cruz Biotechnology Inc. (Dallas, TX, USA): anti-EGFR (1005), anti-AKT (H-136), anti-ERK (K-23), and horseradish peroxidase (HRP)-conjugated goat-anti-rabbit IgG, goat-anti-mouse IgG, and donkey-anti-goat IgG. Quercetin (CAS 6151-25-3) and cycloheximide were purchased from Sigma-Aldrich (St. Louis, MO, USA). The purity of quercetin was at least 95%, as determined by high-performance liquid chromatography analysis. Brigatinib was purchased from Selleckchem (Houston, TX, USA).

### 2.2. Cell Line and Culture

A549 cells, H1975 cells, and HFBs were purchased from the American Type Culture Collection (Manassas, VA, USA). H1975-MS35 cells carrying EGFR C797S were generated by CRISPR/Cas9 knock-in, as described previously [7]. The sequencing chromatograms of C797S mutation in H1975-MS35 are shown in Supplementary Figure S1. Short tandem repeat profiling was used to verify the identity of all cell lines.

### 2.3. Plasmids and Transfection

Plasmids expressing human AXL were constructed by cloning the cDNA of AXL into the pcDNA3.1 vector. The transfection of plasmid DNA was performed as previously described [17,18].

### 2.4. Cell Viability Assays

Viability was assayed by staining with trypan blue and/or MTT, as described previously [19]. Combination index (CI) values were calculated to define the synergistic or additive effects of treatment as previously described [20].

### 2.5. Colony Formation Assay

Cells were seeded in 6-well plates (500 cells/well) and incubated for 24 h. Cells were treated with the drug for 24 h and then cultured in the absence of the drug for an additional 6 days. Foci formation was determined by crystal violet staining.

### 2.6. Apoptosis Assays

The induction of apoptosis was evaluated by assessing PARP cleavage using Western blot analysis and/or by detecting phosphatidylserine exposure with Annexin V-FITC using an Annexin-V-FITC apoptosis detection kit (BD Biosciences, Franklin Lakes, NJ, USA) by flow cytometry [21,22].

### 2.7. Western Blot Analysis

Western blotting was performed as previously described in this publication [18].

### 2.8. Real-Time Reverse Transcriptase Polymerase Chain Reaction (RT-PCR)

RNA extraction and RT-PCR were performed as previously described [18,23]. In brief, RNA was extracted using the TRIzol reagent (Invitrogen, Carlsbad, CA, USA) and used for reverse transcription (RT) by the reverse transcription kit (Applied Biosystems, Foster City, CA, USA) according to the manufacturer’s instructions. The primers used for detecting AXL were AXL-F: CGTAACCTCCACCTGGTCTC and AXL-R: TCCCATCGTCTGACAGCA. The primers used for detecting the mRNA of glyceraldehyde 3-phosphate dehydrogenase (GAPDH) and the condition for PCR amplification were as previously described [23].

### 2.9. Xenograft Mouse Model

H1975-MS35 cells (2 × 10^6^) were subcutaneously injected into the flanks of six-week-old male Balb/c nude mice (NARLabs, Taipei, Taiwan). When the tumors formed from injected cells had grown to approximately 40 mm^3^, mice were randomly allocated into groups of four animals to receive the following treatments: (i) vehicle control, (ii) 25 mg/kg brigatinib once daily by oral gavage, (iii) 50 mg/kg quercetin by intraperitoneal injection or (iv) a combination of (ii) and (iii). Tumor sizes and body weights were measured three times a week, and the tumor volumes were calculated as follows: volume = 0.5 × (length) × (width) ^2^. At the end of the study, mice were sacrificed by CO_2_ asphyxiation, and tumors were harvested to determine the tumor weight and for histology analysis. All animal studies were performed following the guidelines for the Animal Care Ethics Commission of Chang Gung University (IACUC approval number: CGU107-034) and Chang Gung Memorial Hospital (IACUC approval number: 2019032009).

### 2.10. Immunohistochemistry (IHC)

IHC was performed, as described previously [23]. In brief, the tumors from the nude mice were fixed in formalin and embedded in paraffin before sectioning. The 2 μm thick sections were fixed in ice-cold acetone and deparaffinized in xylene, rinsed in alcohol, and then treated with 3% hydrogen peroxide to block endogenous peroxidase. After rinsing in distilled water, antigen retrieval was carried out by boiling in 1 × Trilogy. The tissue sections were incubated first with primary antibody against AXL, phospho-EGFR, phospho-STAT3, or cleaved caspase 3 and then with the secondary antibody. A brown color was developed with AEC substrate chromogen (Dako Corporation, Santa Clara, CA, USA). The computerized quantitation of the target proteins was carried out by using Nikon Br imaging processing software (NIS Basic Research, Version 3.1, Tokyo, Japan) [24].

### 2.11. Molecular Docking Analysis

Docking analysis of AXL with quercetin was performed using BIOVIA Discovery Studio v19.1.0.18287, as described previously [25]. In brief, the 3D crystallographic structures of AXL (PBD ID: 5U6B) were obtained from the Protein Data Bank (PDB). The structure of quercetin was downloaded from the PubChem website. BIOVIA Discovery Studio was used to prepare the AXL structure by removing water and ligand to obtain the extract structure. Docking was performed using the BIOVIA Discovery standard protocol. When AXL and quercetin were docked, the docked complexes were visualized.

### 2.12. Statistical Analysis

The presented results are shown as the mean ± SD of three independent experiments. Statistical analysis was performed by using Student’s *t*-test or by One-Way ANOVA (Analysis of Variance) for comparison of multiple-groups. The *p*-values of significance were presented at <0.05 (∗), <0.01 (∗∗), or <0.001 (∗∗∗), as presented.

## 3. Results

### 3.1. Effects of Quercetin on the Viability and Growth of Human NSCLC Cells

**Figure 1:**
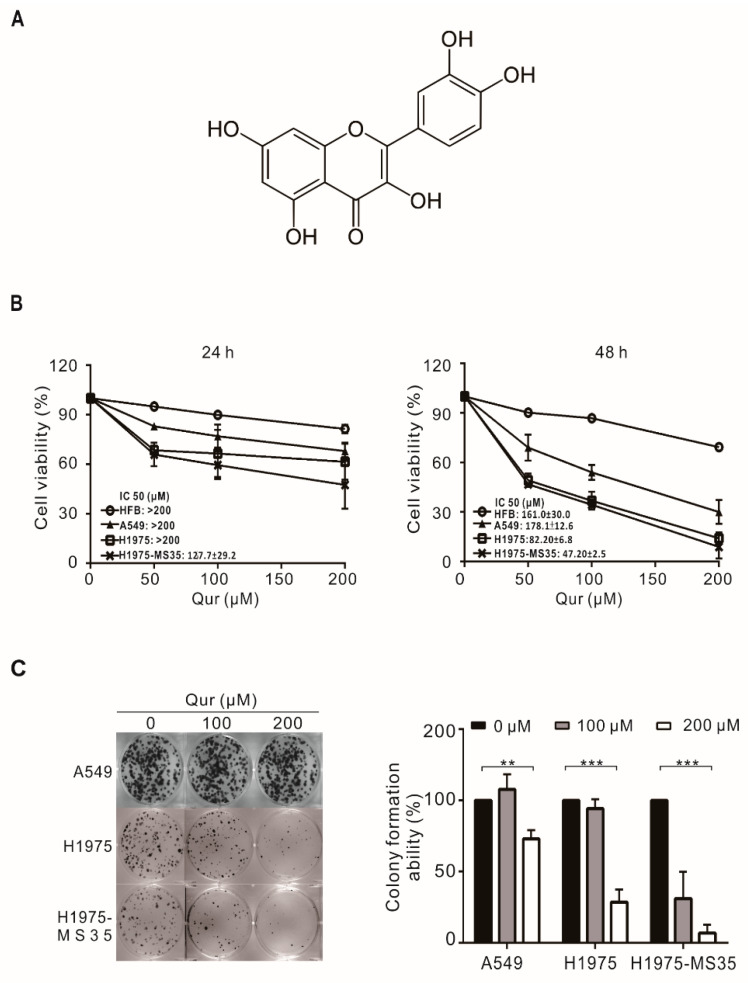
Effects of quercetin on the viability and clonogenic potential of NSCLC cells. (**A**) The chemical structure of quercetin. (**B**) NSCLC cells and normal human fibroblasts (HFBs) were treated with 0–200 μM quercetin (Qur) for 24 or 48 h, and viability was determined with trypan blue assays. (**C**) NSCLC cells were treated with 0–200 μM quercetin for 24 h and cultured for an additional 6 days in the absence of the drug. The colonies were counted and evaluated to determine the relative colony formation ability. The data are expressed as the mean ± SD of three independent experiments. Symbols: ** *p* < 0.01 and *** *p* < 0.001 as analyzed by One-Way ANOVA.

To evaluate the feasibility of using quercetin (Figure 1A) in the treatment of TKI-resistant NSCLCs, we examined the cytotoxic effects of quercetin on NSCLC cells, including A549 (wild-type EGFR), H1975 (EGFR L858R^+^T790M) and H1975-MS35 (EGFR L858R^+^T790M^+^C797S) cells. H1975 cells are sensitive to third-generation TKIs (AZD9291), while the acquisition of the EGFR C797S mutation in H1975-MS35 renders the cells resistant to AZD9291 treatment [7]. As shown in Figure 1B, while quercetin treatment exhibited little or no cytotoxic effect on normal human fibroblasts (HFBs), quercetin reduced the viability of human NSCLC cells in a time- and concentration-dependent manner, suggesting that the cytotoxic effect of quercetin is selective for NSCLC cells. NSCLC cells carrying activating EGFR mutations (H1975-MS35 and H1975) appeared to exhibit higher sensitivity to quercetin than A549 cells (Figure 1B). Next, we examined the effect of quercetin on the colony-forming ability of NSCLC cells. As shown in Figure 1C, the colony-forming ability was suppressed to a much greater extent in H1975 and H1975-MS35 cells than in A549 cells. Together, these results suggest that quercetin exhibits greater cytotoxicity in NSCLC cells harboring EGFR mutations.

### 3.2. Effects of Quercetin on the Induction of Apoptosis and Autophagy in NSCLC Cells

**Figure 2:**
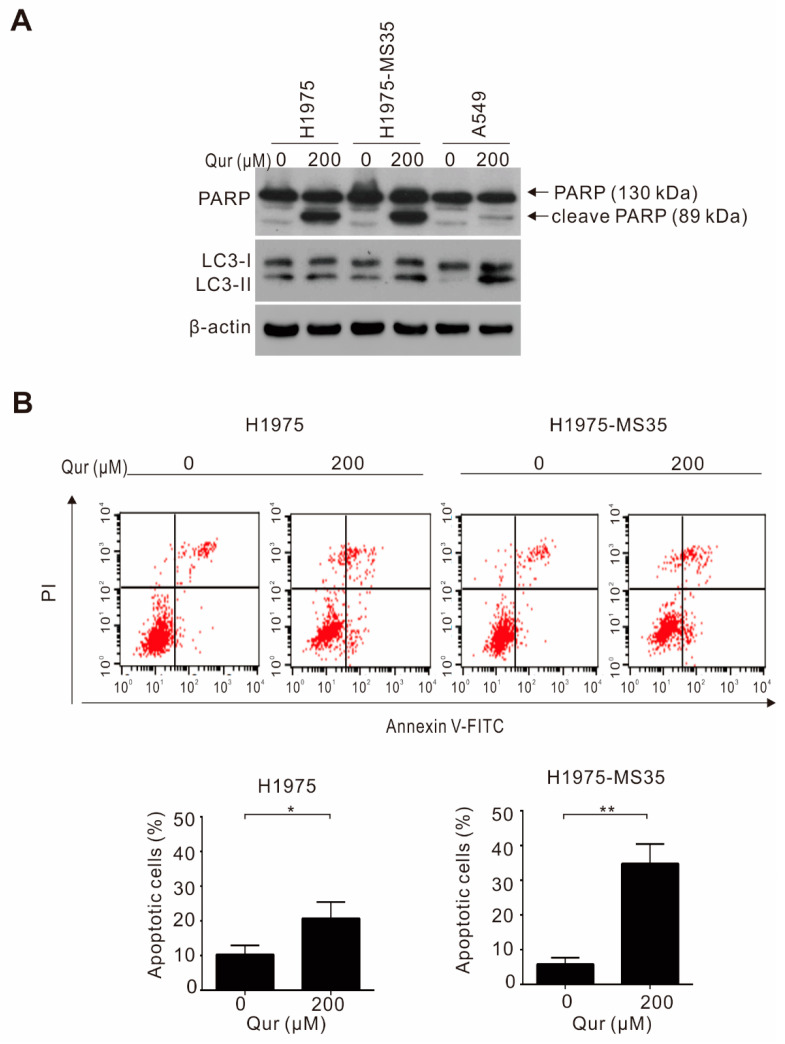
Effects of quercetin on apoptosis and autophagy induction in NSCLC cells. (**A**) NSCLC cells were treated with quercetin for 24 h. The levels of LC3-I, LC3-II, and cleaved PARP (cl-PARP) were determined by Western blotting. β-Actin served as the loading control. (**B**) H1975 and H1975-MS35 cells were treated with quercetin for 24 h, and the induction of apoptosis was assayed by the detection of phosphatidylserine exposure with Annexin V-FITC using flow cytometry. Symbols: * *p* < 0.05 and ** *p* < 0.01, as analyzed by unpaired *t*-tests.

To address whether the cytotoxic mechanism of quercetin is mediated through the induction of apoptosis and/or autophagy, NSCLC cells (H1975, H1975-MS35, and A549) were treated with quercetin and examined for apoptosis induction by the detection of PARP cleavage and for autophagy by the detection of the autophagy marker LC3-II using Western blot analysis. As shown in Figure 2A, the level of cleaved PARP was greatly increased in quercetin-treated H1975 and H1975-MS35 cells compared to quercetin-treated A549 cells. The autophagy marker LC3-II was not detected in untreated A549 cells but was detected in untreated H1975 and H1975-MS35 cells. Treatment with quercetin greatly increased the level of LC3-II in A549 cells, but few changes were detected in the treated H1975 and H1975-MS35 cells. These results suggest that quercetin induces cell death mainly through apoptosis in H1975 and H1975-MS35 cells but largely through autophagy in A549 cells. To determine the extent of apoptosis induction, H1975 and H1975-MS35 cells were incubated with quercetin for 24 h, and apoptosis was detected by flow cytometry with Annexin V-FITC staining. As shown in Figure 2B, the percentages of apoptotic cells (i.e., the cells in the right quadrants of Figure 2B upper panel) among quercetin-treated H1975 and H1975-MS35 cells were 20.6 ± 4.79% and 34.8 ± 5.66%, respectively. Consistent with the results shown in Figure 1C, H1975-MS35 cells were more sensitive to quercetin than H1975 cells.

### 3.3. Quercetin Downregulates the Expression of AXL in EGFR-TKI-Resistant Cells

**Figure 3:**
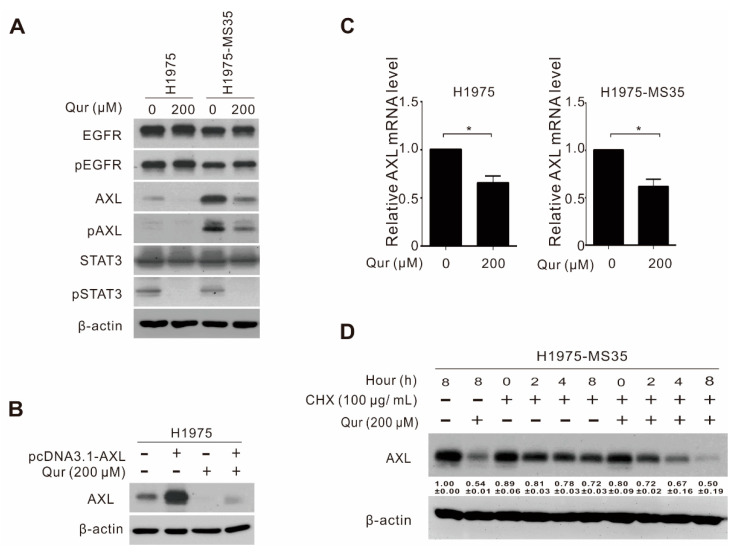
Inhibition of AXL by quercetin in NSCLC cells. (**A**) H1975 and H1975-MS35 cells were treated with quercetin for 24 h, and the cell lysates were assayed for AXL, EGFR, STAT3, phospho (p)-AXL, (p)-EGFR, and (p)-STAT3 expression by Western blotting. β-Actin served as the loading control. (**B**) H1975 cells were transfected with pcDNA3.1-AXL or with pcDNA3.1. After 48 h, the transfected cells were treated with 200 μM quercetin for 24 h, and the levels of AXL were assessed by Western blotting. β-Actin served as the internal control. (**C**) H1975 and H1975-MS35 cells were treated with quercetin for 24 h, and the levels of AXL mRNA were determined by real-time RT-PCR. The expression of AXL mRNA was normalized to that of the untreated cells and is presented as relative expression levels. The data shown are presented as the mean ± SD values. Symbols: * *p* < 0.05 as analyzed by unpaired *t*-tests. (**D**) H1975-MS35 cells were incubated with cycloheximide (CHX) for the indicated times in the absence or presence of quercetin. The relative expression levels of AXL were quantified and are shown at the bottom. β-Actin served as the internal control. The data are expressed as the mean ± SD of three independent experiments.

AXL is a potential driver of various cellular processes, including tumor proliferation, metastasis, and resistance to targeted therapies [26]. As cells carrying the EGFR C797S mutation are associated with the higher expression of AXL [7] and quercetin was reported to inhibit the AXL-STAT3 pathway in glioblastoma cells [16], we hypothesized that quercetin-induced apoptosis and cytotoxicity may be highly related to the inhibition of AXL in H1975 and H1975-MS35 cells. To test this hypothesis, H1975-MS35 and H1975 cells were treated with quercetin and examined for the expression of AXL. As shown in Figure 3A, the treatment of H1975-MS35 and H1975 cells with quercetin reduced the levels of AXL and phosphorylated AXL (pAXL) but had no effect on the levels of total EGFR and phosphorylated EGFR (pEGFR). In addition, we confirmed that quercetin reduced the level of phosphorylated STAT3 (pSTAT3) in H1975 and H1975-MS35 cells. To further confirm that quercetin is involved in modulating AXL expression in NSCLC cells, we transfected the pCDNA3.1-AXL plasmid into H1975 cells and examined whether the ectopically expressed AXL in the transfected cells was sensitive to quercetin inhibition. As shown in Figure 3B, transfection of the AXL expression plasmid into H1975 cells efficiently increased the expression level of AXL. Treatment with quercetin reduced the level of AXL in both the control and AXL-transfected cells.

To address whether the inhibition of AXL expression by quercetin in NSCLC is due to the transcriptional inhibition of AXL or by affecting AXL protein stability, we examined the mRNA expression of AXL and performed protein stability analysis of AXL. As shown in Figure 3C, the real-time RT-PCR results showed that the level of AXL was decreased in quercetin-treated H1975 and H1975-MS35 cells. To determine whether the reduced AXL expression might result from enhanced degradation, H1975-MS35 cells were treated with cycloheximide in the absence or presence of quercetin, and the level of AXL was detected by Western blotting. As shown in Figure 3D, the expression level of total AXL decreased slowly in the absence of quercetin treatment. However, the level of AXL was rapidly reduced in quercetin-treated H1975-MS35 cells, indicating that quercetin affects the stability of AXL. These results indicate that quercetin downregulates AXL both at the transcriptional level and at the posttranslational level in NSCLC cells, consistent with the finding in glioblastoma cells [16].

### 3.4. The Effects of Quercetin and Brigatinib on the Growth of H1975-MS35 Tumor Cells In Vitro and In Vivo

**Figure 4:**
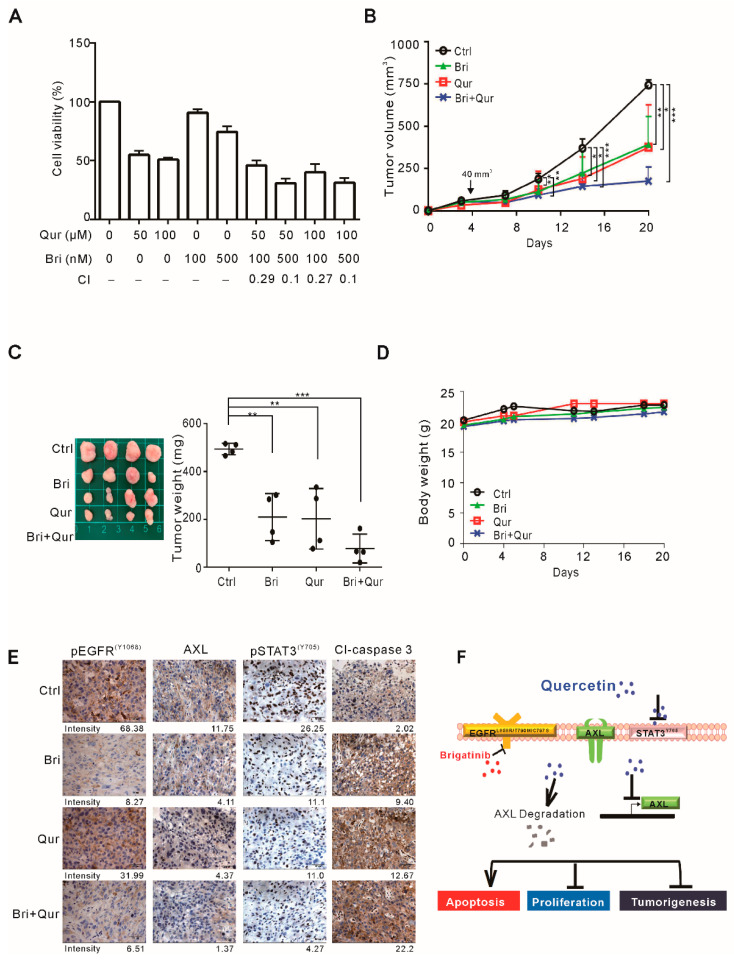
Effects of quercetin and brigatinib on the growth of H1975-MS35 cells in vitro and in vivo. (**A**) H1975-MS35 cells were treated with various concentrations of quercetin and/or brigatinib for 24 h. The viability of the treated cells was determined with trypan blue staining assays. The data are presented as the mean ± SD. Combination index (CI) values are shown at the bottom. (**B**–**D**) H1975-MS35 cells were injected subcutaneously into the flank of each mouse. When the tumor volumes reached approximately 40 mm^3^, the mice were treated with vehicle control, 25 mg/kg brigatinib, or/and 50 mg/kg quercetin once daily (n = 4 per group). The tumor volumes were measured three times a week and are shown in (**B**). The tumors were excised from the mice at the end of the experiment (20 days) and are shown in (**C**). The body weight of treated mice is shown in (**D**). (**E**) Immunohistochemical staining for AXL, phospho-EGFR (pEGFR), phospho-STAT3 (pSTAT3) and cleaved caspase 3 (Cl-caspase 3). The average intensity of the target proteins in IHC are shown below each picture. The results shown in (**B**–**D**) are presented as the means ± SD of four mice. Symbols: * *p* < 0.05; ** *p* < 0.01; and *** *p* < 0.001 by One-Way ANOVA. (**F**) Schematic presentation summarizing the putative action of quercetin as an inhibitor in NSCLC cells harboring EGFR-L858R/T790M/C797S.

To explore a suitable treatment method for EGFR C797S-mediated TKI resistance, we examined the effects of quercetin and brigatinib on tumor growth in vivo. Brigatinib, a dual-target inhibitor of EGFR and anaplastic lymphoma kinase, was reported to overcome AZD9291 resistance presented with EGFR C797S in lung cancer [27,28]. The efficacy of brigatinib and its combination with quercetin for the treatment of EGFR C797S-mediated TKI resistance was first examined with cultured H1975-MS35 cells in vitro. As shown in Figure 4A, although treatment with brigatinib at 100–500 nM had mild cytotoxic effects, the combination of this drug with quercetin produced a synergistic effect on the treatment outcome, suggesting that this is a good combination. However, when similar study was conducted with A549 and H1975 cells, we observed that the combination of brigatinib with quercetin did not produce synergistic cytotoxicity on these two cell lines (Supplementary Figure S2).

To test whether this combination may be useful for treatment in vivo, we used a xenograft mouse model to evaluate the antitumor activity. As shown in Figure 4B,C, treatment with quercetin and brigatinib significantly reduced tumor growth, whereas there was no obvious sign of cytotoxicity or noticeable alteration in body weight (Figure 4D). Consistent with the results of the in vitro study, the combination of quercetin and brigatinib exhibited synergistic antitumor activity against NSCLC cells harboring the EGFR C797S mutation in vivo. To evaluate the inhibition of xenograft growth by quercetin and brigatinib, the levels of AXL, phospho (p)-STAT3, phospho (p)-EGFR, and cleaved caspase 3 were examined in the excised tumor xenografts. As shown in Figure 4E, the levels of AXL and pSTAT3 but not pEGFR were reduced in quercetin-treated tumors. In contrast, the level of pEGFR was reduced in brigatinib-treated tumors, while the levels of AXL and pSTAT3 were not reduced. The cleavage of caspase 3 was increased in tumor xenografts treated with either quercetin or brigatinib and was greatly increased in tumors treated with both quercetin and brigatinib. These results are consistent with the model in which quercetin inhibits tumor growth by suppressing the expression of AXL and activation of STAT3 in NSCLC cells, while brigatinib inhibits tumor growth by suppressing pEGFR in NSCLC cells (Figure 4F).

## 4. Discussion

Quercetin was demonstrated to exert antitumor activity in NSCLCs harboring wild-type EGFR [15,29]. In this study, we demonstrated that quercetin exhibited a greater cytotoxic effect in NSCLC cells harboring TKI-resistant EGFR mutations than wild-type EGFR (Figure 1). The greater cytotoxic effects of quercetin observed in TKI-resistant cells appeared to correlate with the extent of apoptosis induction, as indicated by the detection of greater PARP cleavage in TKI-resistant cells (Figure 2A). As quercetin was shown to induce autophagy to promote apoptosis in A549 cells [29], we examined whether apoptosis induction may be related to autophagy induction. Consistent with a previous study, we observed that quercetin increased the expression of LC3II, an autophagy marker, in quercetin-treated A549 cells (Figure 2A). However, an increase in LC3-II was not detected in quercetin-treated NSCLC cells harboring activating EGFR mutations. These results suggest that quercetin-induced apoptosis in NSCLC cells harboring activating EGFR mutations is independent of autophagy induction.

Quercetin was reported to induce apoptosis by inhibiting the AXL-STAT3 axis pathway in glioblastoma cells [16]. Since the acquisition of the EGFR-C797S mutation resulted in the upregulation of AXL [7] and cells harboring C797S (H1975-MS35) were more sensitive to quercetin than the parental H1975 cells (Figure 1 and Figure 2), we hypothesized that apoptosis induction by quercetin in H1975-MS35 cells may be related to the inhibition of AXL. As shown in Figure 3A, quercetin downregulated the expression of AXL in both H1975 and H1975-MS35 cells. As shown in Figure 3C,D, the inhibition of AXL occurred at both the transcriptional and posttranscriptional levels. Hypoxia-inducible factor 1α (HIF-1α) and activator protein 1 (AP1) are known to be involved in modulating the transcriptional expression of AXL [30]. As quercetin is a potent inhibitor of AP1 and HIF-1α [31,32,33], it is likely that quercetin may affect AXL transcription by inhibiting AP1 and HIF-1α.

To understand how quercetin may affect the posttranslational decreases in AXL, we performed a molecular docking analysis. Quercetin was docked into the crystal structure of the AXL protein (PDB ID: 5U6B), and the docking analysis results were visualized with Discover Studio. The docking analysis showed docking potential with the kinase domain of AXL (Supplementary Figure S3A). Based on the bonding distance, bonding type, and the position of quercetin, Lys567, Pro621, Arg676, and Asp690 of AXL could be important binding sites for forming hydrogen bonds with quercetin (Supplementary Figure S3B). It is suggested that such an interaction of quercetin with AXL could lead to a conformational change in AXL and render it more susceptible to degradation.

As quercetin exerts substantial cytotoxic effects on cultured NSCLC cells harboring the EGFR C797S mutation, we examined whether quercetin could inhibit the growth of such cells in a nude mouse model. As shown in Figure 4B,C, quercetin inhibited tumor growth to a similar extent to brigatinib, a drug known to overcome AZD9291 resistance associated with EGFR C797S in lung cancer [27,28]. Interestingly, the combination of brigatinib and quercetin produced enhanced antitumor activity. The synergistic inhibition by brigatinib and quercetin is likely attributed to the different inhibitory pathways of these drugs, i.e., brigatinib inhibits EGFR, while quercetin inhibits AXL and STAT3 (Figure 4E,F). In summary, herein, we demonstrated that quercetin is a potential therapeutic and/or adjuvant agent for the treatment of NSCLC harboring EGFR-L858R/T790M/C797S mutations.

Finally, it should be pointed out that the results reported in this study were obtained from only one clone (H1975-MS35). It is possible that clonal variation may produce somewhat different outcomes. At present, however, we have only one clone (H1975-MS35) that is confirmed to harbor EGFR L858R, T790M, and C797S triple mutations [7]. Therefore, we are not able to examine the issue about clonal variation.

## 5. Conclusions

Quercetin is an effective inhibitor for the treatment of non-small-cell lung cancer harboring the EGFR C797S mutation.

## Data Availability

The data presented in this study are available on request from the corresponding author.

## Supplementary Materials

The following are available online at https://www.mdpi.com/article/10.3390/biom11091271/s1, Figure S1: The sequencing chromatograms of C797S mutation in H1975-MS35. Figure S2: Effects of quercetin and brigatinib on the growth of H1975 and A549 cells. Figure S3: A docking model of AXL and quercetin.
