# Supplementary material for: Growth Suppression in Lung Cancer Cells Harboring EGFR-C797S Mutation by Quercetin
Source: Biomolecules. 2021 Aug 25;11(9):1271. doi: 10.3390/biom11091271 (PMC8470952; doi:10.3390/biom11091271)
Supplement: Supplementary file 1 [file biomolecules-11-01271-s001.zip › biomolecules-1337599-supplementary.pdf]

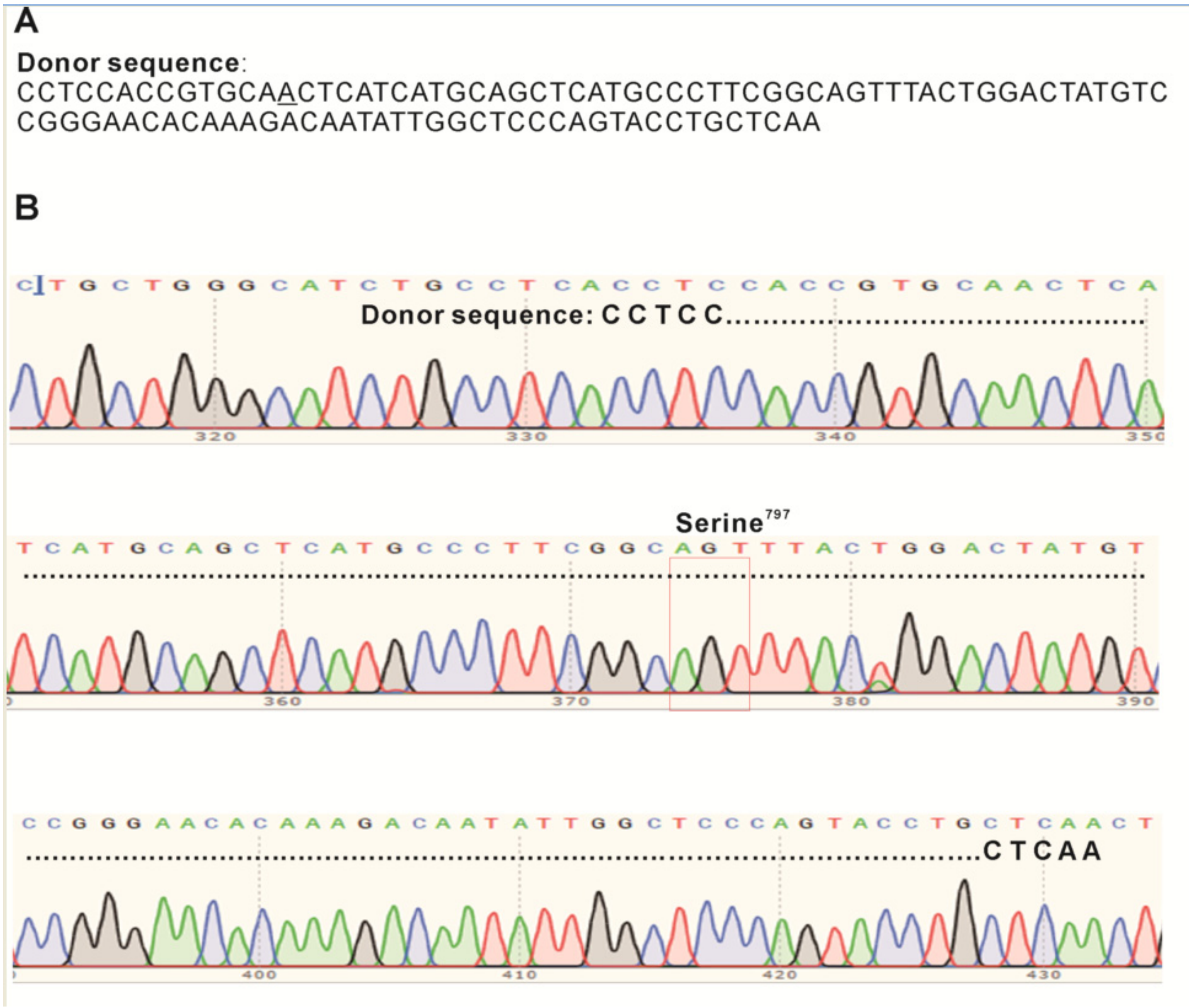

Figure S1. (**A**)The donor DNA, which contains the mutation of EGFR C797S and its right and left homologous arms, was used to replace the C797 sequence via homology-directed repair. (**B**) Sequencing chromatograms of the 100-nt donor DNA region in H1975-MS35 cells.

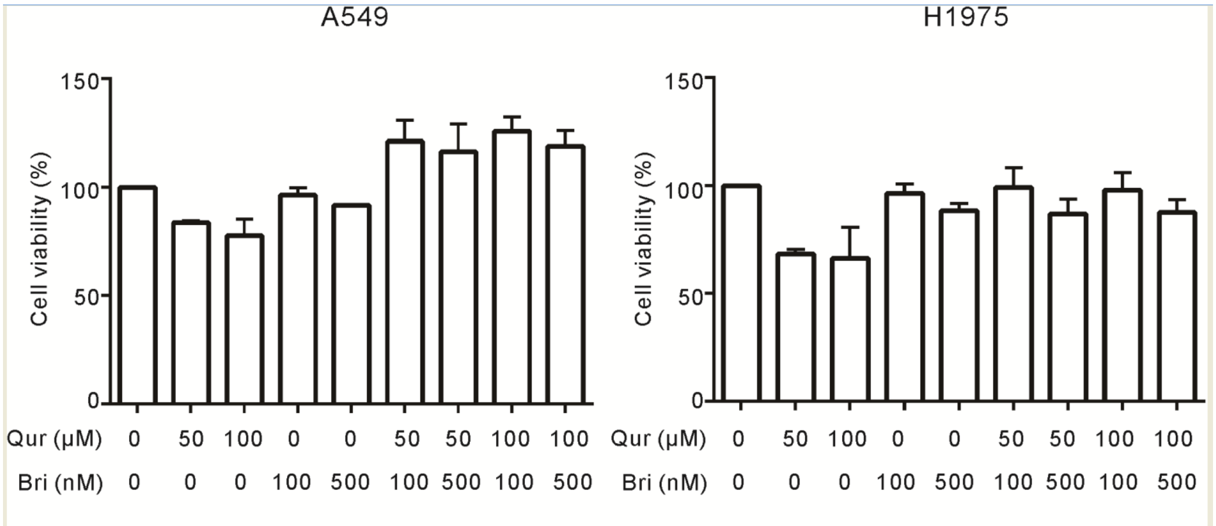

Figure S2. Effects of quercetin and brigatinib on the growth of H1975 and A549 cells. Cells were treated with various concentrations of quercetin and/or brigatinib for 24 h. The viability of the treated cells was determined with trypan blue staining assays. The data are presented as the mean ± SD.

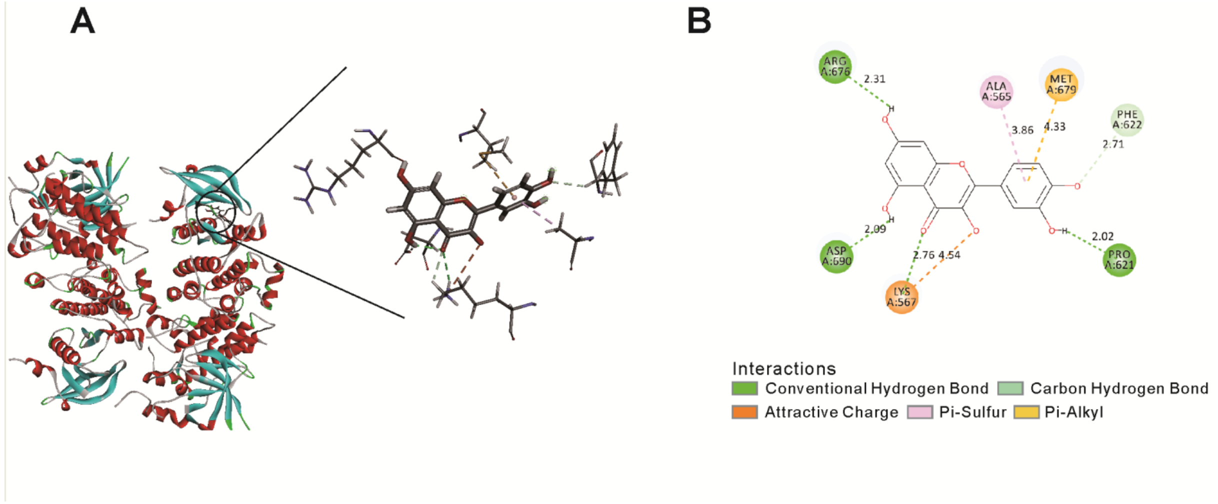

Figure S3. (**A**) The binding residues of quercetin and AXL. (**B**) Two-dimensional diagram showing the conventional hydrogen bond to Lys567, Pro621, Arg679, and Asp690(green dashed lines), and the Pi-alkyl interaction to Ala565(the pink dashed lines), and Pi-sulfur interaction toMet679(orange dashed lines).
